# Concurrent involvement of the bone marrow by BRAF V600E–mutant melanoma and hairy cell leukemia

**DOI:** 10.1007/s12308-024-00609-3

**Published:** 2024-10-28

**Authors:** Margaret Moore, Pranav Patel, Jianguo Tao

**Affiliations:** 1 Department of Pathology and Laboratory Medicine, University of Virginia, Charlottesville, VA USA; 2 Division of Hematology and Oncology, University of Virginia, Charlottesville, VA USA

**Keywords:** Hairy cell leukemia, Melanoma, Targeted therapy

A 49-year-old female with stage IV melanoma was noted to have a MCV of 100.6 fL, absolute neutrophil count of 1.39 K/µL, and platelets of 109 K/µL, as part of laboratory evaluation during routine oncology care. Additional CBC indices, including absolute lymphocyte count, were unremarkable. A bone marrow biopsy was performed to evaluate the cytopenias. At the time of biopsy, the patient had FDG-avid bone lesions by PET/CT, with biopsy-confirmed metastatic melanoma within the humerus. She had recently received two cycles of ipilimumab and nivolumab. Splenomegaly was not identified by physical exam or imaging.

The bone marrow aspirate demonstrated melanoma cells with vacuolated cytoplasm, eccentric nuclei, and prominent nucleoli (Fig. 1a). Small lymphocytes with fine, circumferential cytoplasmic projections were also noted. The core biopsy showed a hypercellular marrow with two atypical populations: (1) nodular foci of melanoma, confirmed by SOX10 immunohistochemistry (IHC), and (2) a diffuse infiltrate of CD20-positive B-cells (Fig. 1b–d). *BRAF* V600E IHC was positive in *both* populations (Fig. 1e); *BRAF* mutation was confirmed by next-generation sequencing. Flow cytometry of the aspirate demonstrated a kappa-monotypic B-cell population with increased side-scatter, expressing CD25, CD103, CD11c, and CD123, with absence of CD5 or CD10 expression (Fig. 2a–f). In addition to confirming involvement by melanoma, a new diagnosis of hairy cell leukemia (HCL) was rendered. Dabrafenib and trametinib were initiated, with partial response and improvement in CBC indices. However, the melanoma subsequently progressed, and the patient died of melanoma-associated complications eight months later. Repeat bone marrow evaluation was never performed.

**Fig. 1:**
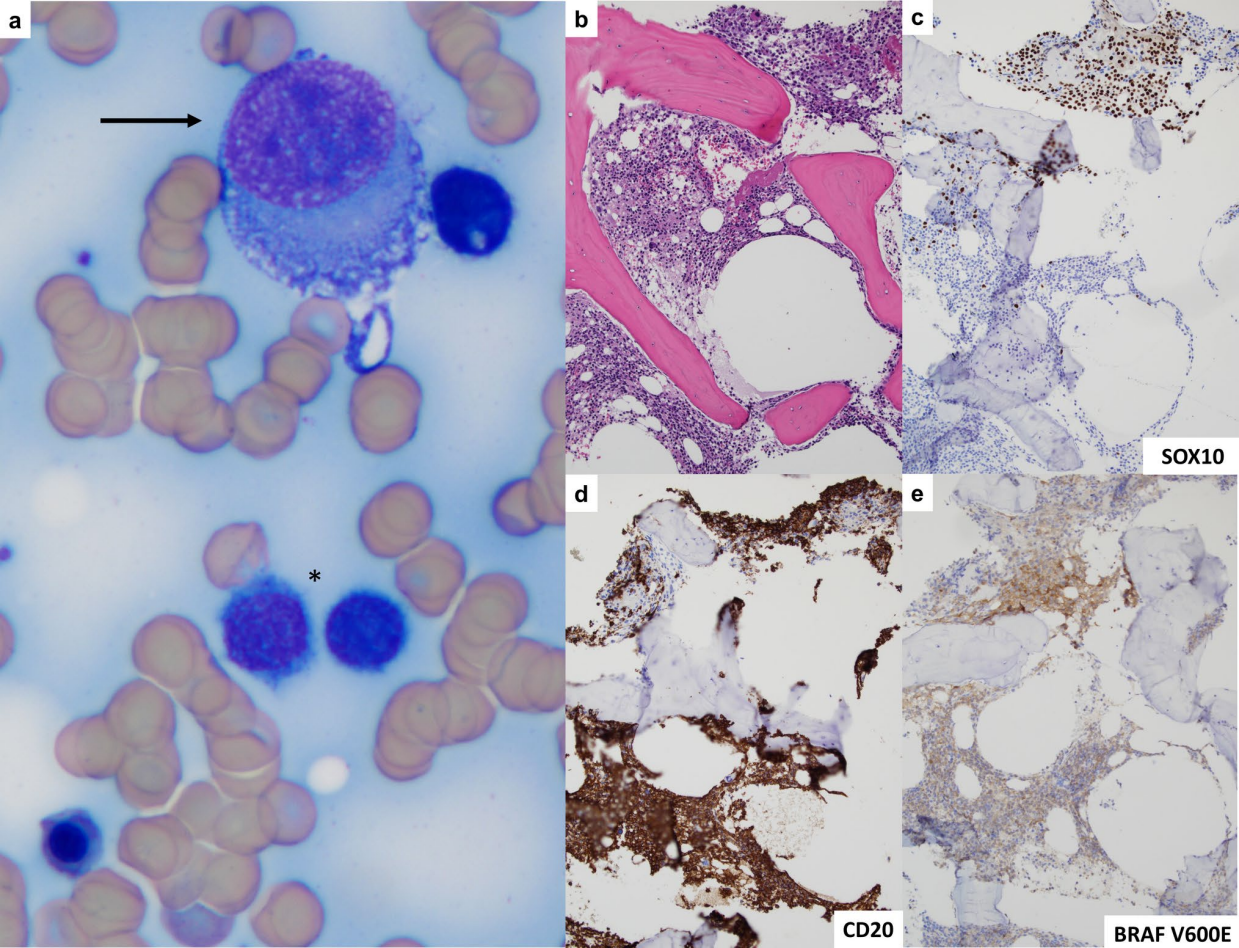
**a** The bone marrow aspirate shows large, atypical melanoma cells with vacuolated cytoplasm (arrow), as well as small lymphocytes with cytoplasmic projections (*). **b**–**e** The core biopsy shows a hypercellular marrow (~ 70% cellular) with an aggregate of melanoma which is positive for SOX10, as well as an interstitial infiltrate of CD20-positive small B-cells. An immunohistochemical stain for BRAF V600E is positive in both the melanoma and B-cell populations

**Fig. 2:**
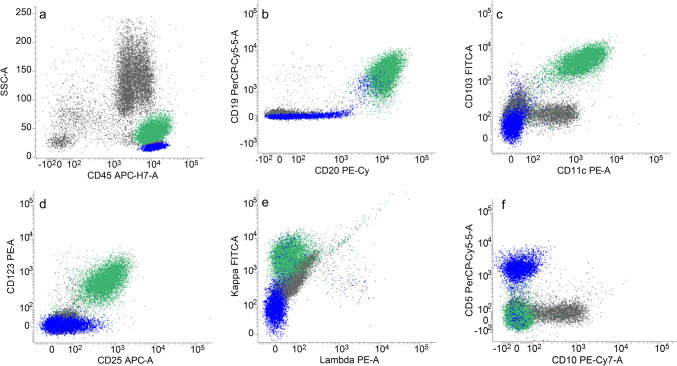
**a** Flow cytometry demonstrates an atypical B-cell population (green) with increased side-scatter compared to background lymphocytes (blue). **b**–**e** This population is positive for CD19, CD20 (bright), CD103, CD11c, CD123, and CD25, with kappa light chain restriction. **f** CD10 and CD5 are negative in the atypical B-cell population

Patients with concurrent (or sequential) BRAF-mutant melanoma and HCL have been described [1, 2]. This is a unique case in which both tumors simultaneously involve the marrow, highlighting the utility of flow cytometry and IHC in delineating the populations. Accurate diagnosis was critical, as the shared presence of *BRAF* mutations provided an opportunity to treat both neoplasms with dabrafenib and trametinib.

## Data Availability

No datasets were generated or analysed during the current study.
